# Correlation of PD-1 and PD-L1 expression in oral leukoplakia and oral squamous cell carcinoma: an immunohistochemical study

**DOI:** 10.1038/s41598-023-48572-w

**Published:** 2023-12-07

**Authors:** L. R. Greeshma, Anna P. Joseph, T. T. Sivakumar, Varun Raghavan Pillai, Gopikrishnan Vijayakumar

**Affiliations:** 1 Department of Oral and Maxillofacial Pathology, PMS College of Dental Sciences and Research, Trivandrum, Kerala India; 2 Department of Oral Maxillofacial Pathology and Microbiology, Pandit Bhagwat Dayal Sharma Post Graduate Institute of Medical Sciences, Rohtak, Haryana 124001 India

**Keywords:** Cancer, Drug discovery, Immunology, Molecular biology, Oncology

## Abstract

The programmed cell death protein (PD-1)/programmed cell death protein ligand (PD-L1) pathway and cytotoxic T lymphocyte antigen are the most important co-stimulatory molecules that play a key role in the negative regulation of T cells during carcinogenesis. We aimed to evaluate the immunohistochemical expression of PD-1 and PD-L1 in oral leukoplakia and squamous cell carcinoma compared with normal oral mucosa. Twenty-five cases of oral squamous cell carcinoma, oral leukoplakia and normal oral mucosa tissue specimens were immunohistochemically stained to assess PD-1 and PD-L1 expression. The PD-L1 positivity of subepithelial TAFs (*p* < 0.001) increased with increasing grades of oral leukoplakia. Pearson’s correlation indicated a high positive correlation between the PD-L1 labelling index of epithelial tumour cells and the PD-1 labelling index of tumour infiltrating lymphocytes (*p* value: 0.005) in OSCC. A high positive correlation was noted between the H-score of PD-L1 positive tumour epithelial cells and the H-score of PD-1 positive tumour infiltrating lymphocytes in OSCC (*p* value: 0.001). PD-L1 positivity increased in dysplastic epithelial cells from premalignant lesions to malignancy. The sub-epithelial PD-L1 positive TAFs were higher in oral leukoplakia compared to OSCC inferring that PD-L1 positivity in TAFs decreased with malignant transformation. The PD-1 positivity in TILs was higher in oral leukoplakia than in OSCC.

## Introduction

Carcinogenesis is a multistep process mediated by various signalling factors that contribute to a significant burden of oral cancer^1,2^. The major risk factor for high prevalence of oral squamous cell carcinoma (OSCC) and its precursor oral potentially malignant disorders is use of various forms of tobacco. Majority of OSCC cases are associated with a clinically evident oral leukoplakia (OL) which is the most common OPMD^3^. Oral leukoplakia shows a worldwide prevalence of 1.5–4.3%, and in India, the prevalence varies from 0.2 to 4.9%^3,4^ with a malignant transformation rate ranging between 5 and 18%^5,6^. The morbidity and mortality rates of OSCC remain high, which could be attributed to advanced stage at diagnosis, and lack of effective therapeutic modalities and biomarkers for early diagnosis and prediction of prognosis^7,8^.

Programmed cell death protein 1 (PD-1/CD279) is a cell surface protein composed of 288 amino acids, which is expressed on surface of activated T-cells, (CD4+ T-cells, CD8+ T-cells, Natural killer cells), B cells and activated monocytes^9^. PD-1/PD-L1pathway and Cytotoxic T lymphocyte antigen 4 (CTLA-4; CD152) are members of the immunoglobulin superfamily which are considered to be the most important co-stimulatory molecules resulting in negative regulation of T cells during carcinogenesis^10,11^. PD-1 has two ligands, PD-L1 (B7-H1; CD274) and PD-L2 (B7-DC; CD273), which are members of B7 family^9,11,12^. PD-L1 is commonly expressed on the surface of tumour epithelial cells, and few other cells including antigen-presenting cells (APCs), T cells, B cells, monocytes and tumour-associated fibroblasts (TAFs)^13^. PD-1 and its ligand PD-L1 are emerging biomarkers of early detection and potential therapeutic target for immune check point inhibitor, against tumour immune escape mechanisms in various carcinomas^7,8^.

The expression of PD-L1 in tumour cells activates the PD-1/PD-L1pathway by binding to the PD-1 receptor on activated T-lymphocytes which leads to inhibition of cytotoxic T cells against cancer, and thus permitting cancer progression and metastasis. These deactivated T-cells remain inhibited in the tumour microenvironment^10,11,14,15^. The PD-1/PD-L1 pathway hence behaves as an adaptive immune resistance mechanism exerted by the tumour cells in response to endogenous anti-tumour activity. Such interaction has an inhibitory and a regulatory role in T-cell responses and maintenance of natural immune tolerance against a self-antigen^16^. Hence, a therapeutic blockade of PD-L1 could enhance the immune response and decrease tumour resistance in a variety of tumours. Such effective therapeutic benefits are observed in tumours like melanoma and non-small cell lung cancer and thus serves as an effective method in treatment and prognosis^11^.

In OSCC, which is often preceded by a pre malignant lesion, various molecular pathways and signalling proteins play a major role in malignant transformations. Identification of PD-1 and PD-L1 pathway in the oral premalignant lesions may lead to newer insights not only for better understanding of its molecular pathogenesis, and malignant transformation, but also for the future treatment modalities. Thus, in the present study we evaluated the immunohistochemical expression of PD1 and PD-L1 in formalin fixed paraffin embedded (FFPE) tissues of oral leukoplakia with dysplasia (OL) and OSCC, and compared the expression with normal oral mucosa (NOM).

## Methods

The present study was conducted in the Department of Oral Pathology and Microbiology, PMS College of Dental Science and Research, Vattapara, Trivandrum after the approval by Institutional Ethics Committee. All experiments were performed in accordance with relevant guidelines and regulations with the 1964 Helsinki declaration and its later amendments or comparable ethical standards.

### Study sample

The study sample comprised a total of 75 cases in which 25 were OSCC, 25 cases of OL (oralleukoplakia with epithelial dysplasia) and 25 cases of NOM. Samples of inflammation free gingival tissues obtained from cases of surgical removal of impacted molars of healthy individuals were taken as NOM. All specimens were procured from routine biopsy specimen in the department during the study period.

### Selection criteria

Patients with only primary OSCC lesions and oral leukoplakic lesions were taken for study. Histologically proven cases from any intraoral site vulnerable for biopsy procedure were considered. Unwilling patients and with systemic diseases or previously treated cases were excluded from the study.

### Immunohistochemistry

**Figure 1:**
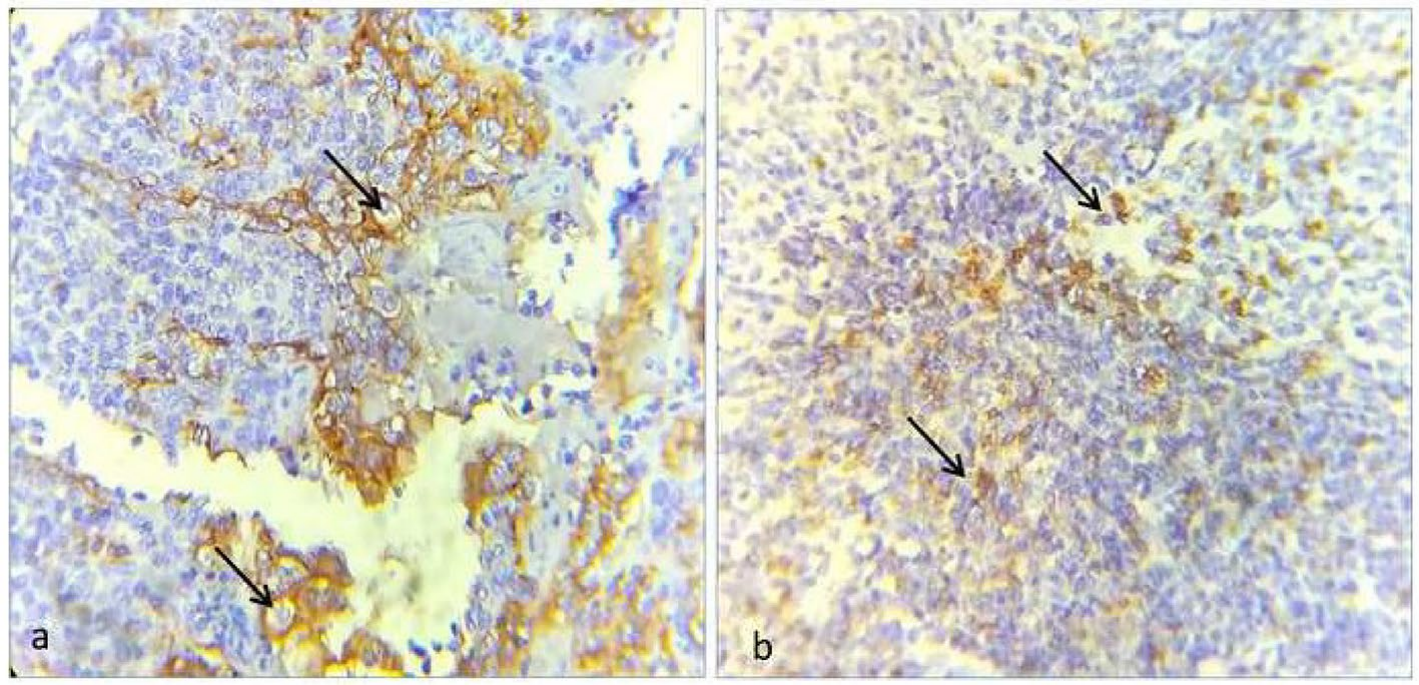
Photo micrograph showing immunohistochemical expression. (**a**) Tonsillar epithelium as positive control of PD-L1 (400×) (black arrow heads). (**b**) Tonsillar lymphocytesas positive control for PD-1 (400×) (black arrow heads).

Formalin fixed paraffin embedded (FFPE) blocks of each, 4-micron thick sections on APES coated slides were subjected to immunohistochemical analysis for PD1 (Anti-human PD-L1 Rabbit Monoclonal Antibody, IgG isotype (CAL10, Master diagnostica (Vitro S.A), Granada, Spain) and PD-L1 (Anti-human PD-1 Mouse Monoclonal Antibody, IgG1 isotype (CAL10, Master diagnostica (Vitro S.A), Granada, Spain). Tissue sections were deparaffinized in xylene (twice), treated with a graded series of alcohol (100%, 95%, 85% and 75% ethanol), and then incubated in phosphate buffered saline (PBS, pH 7.4) for 5 min. Heat induced antigen retrieval was done by immersion in 10 mM Tris–EDTA with pH 9 at 600 W in 5 L capacity pressure boiler until two whistles. Endogenous peroxidase was inactivated by 3% hydrogen peroxide for 10 min. The tissue sections were incubated with primary antibodies against PD1 and PD-L1 overnight in moist chamber. After wash with PBS, a target enhancer was used (Poly excel target binder -Prediluted IPS006, 6 mL) to cover the section followed by incubation in moist chamber at room temperature for 12–20 min. The slides were incubated for 20 min in humidifying chamber with secondary polyclonal conjugate (Poly excel poly HRP anti-rabbit -prediluted PEH002, IPSS007, Pathinsitu, India). Lastly, tissue sections were treated with diaminobenzidine as a substrate chromogen and counterstained with haematoxylin. As negative controls, tissue sections were treated with phosphate buffered saline instead of the primary antibody. Tonsillar epithelium and tonsillar lymphocytes were taken as positive controls for PD-1 and PD-L1 respectively (Fig. 1a,b) The slides were then mounted, observed and evaluated under a light microscope.

### Evaluation of immunohistochemical stain

PD-L1 and PD-1 expressions were graded according to criteria by Maruse et al.^11^, Cho et al.^17^, Kitano et al.^18^, Yagyuu et al.^19^, Fiedler et al.^20^ and de Ruiter et al.^21^. Each immunoassayed section was independently analysed by two experienced pathologists. Three randomly selected fields in each slide were evaluated at magnification of 200×. Each area was selected to include at least 100 cells and value derived by two pathologists were averaged for a final result. And also Bland Altman analysis was performed to study the interobserver variability, and this was found to be agreeable.

#### Evaluation of PD-L1 in OSCC and oral leukoplakia

In OSCC, PD-L1 expressed in tumour epithelial cells and tumour associated fibroblasts (TAFs) were counted. In oral leukoplakia, PD-L1 expressed in dysplastic epithelial cells and tumour associated fibroblasts in underlying connective tissue (TAFs) were counted. For scoring the staining in epithelial cells, the labelling index and H score was used and for PD-L1 positivity in tumour associated fibroblasts (TAFs), the staining intensity, labelling index and staining intensity–distribution score (SID) was assessed. Epithelial cells with complete circumferential or partial linear plasma membrane or cytoplasmic staining and tumour associated fibroblast (TAFs) with complete cytoplasmic and membrane staining was considered as positively stained (brown reaction product).

##### Labelling index

Labelling index = Number of PD-L1 positive tumour epithelial cells/Total number of viable tumour cells × 100.

##### H-score

H score = 0 × % of non-stained tumour epithelial cells + 1 × % of weakly stained tumour epithelial cells + 2 × % of moderately stained tumour epithelial cells + 3 × % strongly stained tumour epithelial cells.

##### Staining intensity of tumour associated fibroblasts (TAFs)

Staining intensity of PD-L1 positive tumour associated fibroblasts (TAFs) was evaluated and compared with the staining intensity of positive control tonsil and scored as: score 0-negative (non-stained cells), score 1-staining expression weaker than that of tonsil-weak, score 2-staining expression equivalent that of tonsil-moderate and score 3-staining expression stronger than that of tonsil-severe.

##### Labelling index of tumour associated fibroblasts (TAFs)

Labelling index of TAFs = No of PD-L1 positive TAFs/Total number of TAFs × 100.

##### Staining intensity–distribution score (SID) of tumour associated fibroblasts (TAFs)

The distribution of PD-L1 positive sub-epithelial TAFs was graded as: score 0 (0%), score 1 (1–50%), and score 2 (51–100%). A staining intensity –distribution score (SID) was calculated from the formula: SID = Score of distribution of stained cells × Score of staining intensity.

PD-L1 expression of TAFs was categorized as positive or negative, according to Kitano et al.^18^, when sum of distribution and intensity scores (range, 0–5) was 3 or higher.

#### PD-1 evaluation in OSCC and oral leukoplakia

Staining of PD-1 in OSCC and leukoplakia was assessed in lymphocytes. In OSCC, the tumour- infiltrating lymphocytes (TILs) within the tumour cell nests (intratumoral) and in the stroma near the invasive front of OSCC (peritumoral) were counted. While in oral leukoplakia, staining of PD-1 was assessed in subepithelial inflammatory T-lymphocytes cells. A case is considered to be PD-1 positive, if a minimum of thirty membranous stained tumour infiltrating lymphocytes (TILs) (≥ 30%) were seen in OSCC and a minimum of ten membranous stained subepithelial inflammatory T-lymphocytes (≥ 10%) were seen in oral leukoplakia. The labelling index and H score for PD-1 expression in TILs were calculated.

##### Labelling index

Percentage of PD-1-stained TILs = No of PD-1 positive TILs/Total no of TILs × 100.

##### H-Score

H score = 0 × % of non-stained TILs + 1 × % of weakly stained TILs + 2 × % of moderately stained TILs + 3 × % strongly stained TILs.

#### Evaluation of PDl-1 and PD-1 in normal oral mucosa (NOM)

PD-L1 and PD-1 expression in epithelial cells and TILs was evaluated in similar pattern as mentioned above.

### Statistical analysis

Results were statistically analysed by entering the findings into Microsoft excel worksheet and compared for statistical significance using SPSS version 25 (IBM, US). Quantitative variables were expressed as mean ± standard deviation and qualitative variables are expressed as percentages. Chi square test was used to assess the significance of difference between expressions of PD-1 and PD-L1 in OSCC and oral leukoplakia. Univariate and bivariate analysis were done. *p* value less than 0.05 was considered to be statistically significant. Comparison of mean value was done using independent sample t test. Correlation between continuous variable was done using Pearson correlation analysis. Strength of correlation was categorized based on John Hopkins interpretation of correlation coefficients.

### Ethical approval

This article does not contain any studies with animals performed by any of the authors. All procedures performed in this study involving human participants were in accordance with the ethical standards of the institutional and/or national research committee and with the 1964 Helsinki declaration and its later amendments or comparable ethical standards.

### Informed consent

An informed and written consent was obtained from all participates prior to study.

## Results

The present study consisted histologically proven 25 cases each of OSCC, OL and NOM. The demographic data of cases in study are shown in Table 1.

**Table 1:** Showing demographic data and site distribution.

Groups	Total no of samples	Males	Females	Age range (years)	Anatomic sites			
					Buccal mucosa	Tongue	Alveolar mucosa	Gingiva
OSCC	25	17	8	25–85	10	9	3	3
Oral leukoplakia (OL)	25	18	7	20–90	9	7	6	3
Normal oral mucosa (NOM)	25	16	8	18–25	0	0	20	5

### Pattern of expression of PD-L1 in oral squamous cell carcinoma

**Figure 2:**
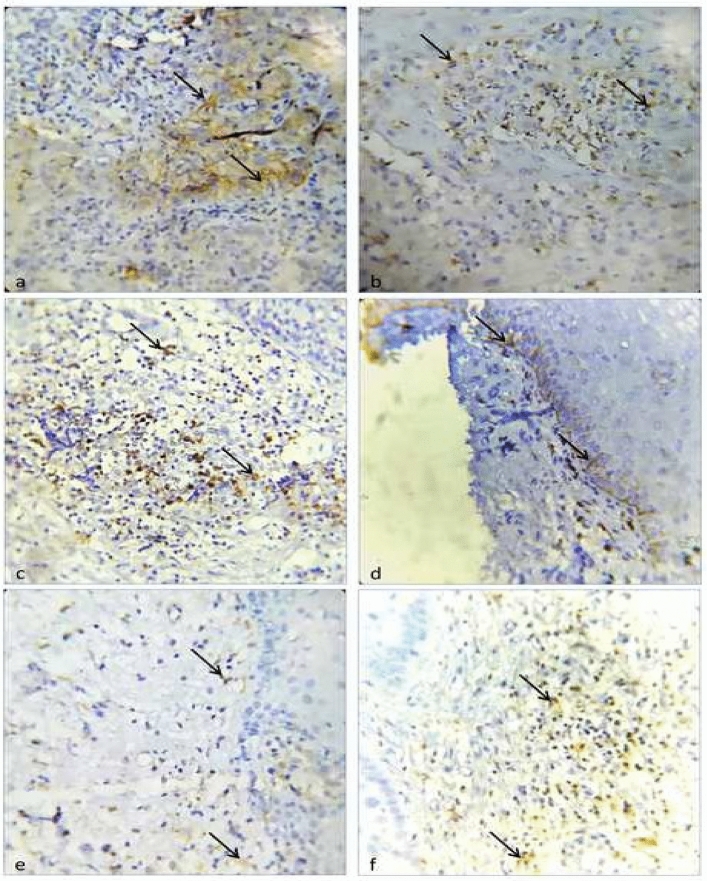
Photo micrograph showing immunohistochemical expression. (**a**) Membranous expression of PD-L1 in epithelial tumour cells present in the invasive front of oral squamous cell carcinoma (400×) (black arrow heads). (**b**) Membranous expression of PD-L1 in tumour associated fibroblast cells present in the invasive front of oral squamous cell carcinoma (400×) (black arrow heads). (**c**) Membranous expression of PD-1 in tumour- infiltrating lymphocytes (TILs) present in the invasive front of oral squamous cell carcinoma (400×) (black arrow heads). (**d**) Membranous expression of PD-L1 in dysplastic basal epithelial cells of oral leukoplakia (400×) (black arrow heads). (**e**) Membranous expression of PD-L1 in tumour associated fibroblast cells present subepithelial stroma of oral leukoplakia (400×) (black arrow heads). (**f**) Membranous expression of PD-1 infiltrating lymphocytes present in subepithelial stroma of oral leukoplakia (400×) (black arrow heads).

Among the 25 samples of OSCC, 11 cases were well differentiated and 14 were moderate/poorly differentiated tumors. About 96% (24/25) cases were PD-L1 positive on calculating the labelling index in tumour epithelial cells (Fig. 2a). On comparing the labelling index between different grades of OSCC, all the 11 cases of well differentiated OSCCs and 13/14 moderate/poorly differentiated OSCCs were PD-L1 positive with a *p* value 0.366 which was statistically non-significant (Table 2).The SID score of PD-L1 positive TAFs was positive in 76% (19/25) cases of OSCC (Fig. 2b) of which 9/11 well and 10/14 moderate/poorly differentiated OSCC showed PD-L1 positive TAFs (*p* value 0.546) which was statistically not significant. The mean H score of PD-L1 positive tumour epithelial cells was more in well than moderate/poorly differentiated OSCC, but the mean difference was not statistically significant.

**Table 2:** Analysis of PD-L1/PD1 in OSCC.

	Well differentiated	Moderately/poorly differentiated	Total	*p* value
Frequency	11	14	25	
PD-L1 positive Tumour epithelial cells (labelling index)	11 (100.0%)	13 (92.9%)	24 (96.0%)	0.366
PD-L1 negative Tumour epithelial cells (labelling index)	0 (0%)	1 (7.0%)	1 (4.0%)	
PD-L1 positive TAF (SID score)	9 (81.8%)	10 (71.4%)	19 (76.0%)	0.546
PD-L1 negative TAF (SID score)	2 (18.2%)	4 (28.6%)	6 (24.0%)	
H score of PD-L1 positive cases (mean ± SD)	69.27 ± 57.446	53.64 ± 36.519		0.416
SID score of TAFs (mean ± SD)	2.00 ± 0.632	1.64 ± 0.225		0.254
PD-1 positive TILs (labelling index)	8 (72.7%)	11 (78.6%)	19 (76%)	0.734
PD-1 negative TILs (labelling index)	3 (27.3%)	3 (21.4%)	6 (24.0%)	
H-score of PD-1 positive TILs (mean ± SD)	73.09 ± 64.689	72.64 ± 55.303		0.985

### Pattern of expression of PD-1 in oral squamous cell carcinoma

About 76% (19/25) cases of OSCC were PD-1 positive, while calculating labelling index of TILs (Fig. 2c). On comparing different grades of OSCC, 8/11 well and 11/14 moderate/poorly differentiated OSCC were PD-1 positive with a *p* value 0.734. The mean H score of PD-1 positive TILs was slightly more in well than moderate/poorly differentiated OSCC, but the mean difference was not statistically significant (Table 2).

### Correlation of expression between PD-L1 and PD 1 in oral squamous cell carcinoma

Correlating the labelling index of PD-L1 positive tumour epithelial cells and labelling index of PD-1 positive tumour infiltrating lymphocytes in OSCC, a Pearson correlation coefficient of 0.544 indicates a high positive correlation which was statistically significant (*p* value: 0.005). Correlating H score of PD-L1 positive tumour epithelial cells and PD-1 positive TILs of OSCC, a Pearson correlation coefficient of 0.606 indicates a high positive correlation which was statistically significant (*p* value: 0.001) (Table 4).

### Pattern of expression of PD-L1 in oral leukoplakia

Among the 25 samples of oral leukoplakia 11 cases were mild dysplasia and 14 were moderate/severe dysplasia. Only 5/25 (20%) showed PD-L1 positivity in dysplastic basal epithelial cells, while 18/25 (Fig. 2d) (72%) cases of oral leukoplakia show subepithelial PD-L1 positivity in TAFs (Fig. 2e). We could observe that 1/11 case of mild dysplasia and 4/14 moderate/severe dysplasia cases exhibited basal PD-L1 positivity which on comparison was statistically non-significant (*p* value 0.227) (Table 3). On comparing the PD-L1 positivity of subepithelial TAFs, 4/11 cases of mild dysplasia and 14/14 moderate/severe dysplasia cases exhibited PD-L1 positivity which was statistically significant (*p* value < 0.001) .The mean SID score of PD-L1 positive TAFs were more in moderate/severe dysplasia compared to mild dysplasia which was found to be statistically significant (*p* value: 0.000).

**Table 3:** Analysis of PD-L1/PD1 in Oral leukoplakia.

	Mild	Moderate/severe	Total	*p* value
Frequency	11	14	25	
PD-L1 positive dysplastic basal epithelial cells (labelling index)	1 (9.1%)	4 (28.6%)	5 (20.0%)	0.227
PD-L1 negative Tumour epithelial cells (labelling index)	10 (90.9%)	10 (71.4%)	20 (80.0%)	
PD-L1 positive TAF (SID score)	4 (36.4%)	14 (100%)	18 (72.0%)	**0.001**
PD-L1 negative TAF (SID score)	7 (63.6%)	0 (0%)	7 (28.0%)	
SID score of PD L1 positive TAFs (mean ± SD)	0.82 ± 1.168	2.86 ± 1.167		**0.000**
PD-1 positive TILs (labelling index)	8 (72.7%)	14 (100%)	22 (88%)	0.037
PD-1 negative TILs (labelling index)	3 (27.3%)	0 (0%)	3 (12.0%)	
H-score of PD-1 positive TILs (mean ± SD)	54.73 ± 73.987	84.57 ± 61.405		0.282

Significant values are in bold.

### Pattern of expression of PD-1 in oral leukoplakia

About 88% (22/25) cases of oral leukoplakia were PD-1 positive, while calculating labelling index of TILs (Fig. 2f). Comparing PD-1 positive cases between the histological grades, 8/11 cases of mild and 14/14 cases of moderate/severe dysplasia were PD-1 positive with statistically significant *p* value (0.037). However, on comparing H-score among histological grades, mean H-score was higher in moderate/severe dysplasia which was statistically non-significant (Table 3).

### Correlation of expression between PD-L1 and PD 1 in oral leukoplakia

Correlating the labelling index of PD-L1 positive TAFs and labelling index of PD-1 positive subepithelial inflammatory T-lymphocytes in oral leukoplakia, the Pearson correlation coefficient of 0.358 indicated a moderate positive correlation which was found to be statistically non-significant (*p* value: 0.079) (Table 4).

**Table 4:** Correlation of expression of PDL1/PD-1 in OSCC and oral leukoplakia.

OSCC		PDL1 positive epithelial cells (labelling index)	PD1 positive lymphocytes (labelling index)
PDL1 positive epithelial cells (labelling index)	Pearson correlation	1	0.544
	Sig (2 tailed)		**0.005**
	N	25	25
PD1 positive lymphocytes (labelling index)	Pearson correlation	0.544	1
	Sig (2 tailed)	0.005	
	N	25	25
PD1 positive lymphocytes (H score)	Pearson correlation	1	0.606
	Sig (2 tailed)		**0.001**
	N	25	25
PDL1 positive epithelial cells (H score)	Pearson correlation	0.606	1
	Sig (2 tailed)	0.001	
	N	25	25

Oral leukoplakia		PDL1 positive TAFs (labelling index)	PD1 positive lymphocytes (labelling index)
PDL1 positive TAFs (labelling index)	Pearson correlation	1	0.358
	Sig (2 tailed)		0.079
	N	25	25
PD1 positive lymphocytes (labelling index)	Pearson correlation	0.358	1
	Sig (2 tailed)	0.079	
	N	25	25

Significant values are in bold.

### Inter group comparison of expression of PD-L1 among OSCC and OL

Comparing epithelial PD-L1 positivity between OSCC and oral leukoplakia, OSCC showed a higher epithelial positivity which was statistically significant (*p* value of < 0.001). On comparing PD-L1 positivity of sub epithelial TAFs between OSCC and oral leukoplakia, OSCCs have a slightly higher positivity than oral leukoplakia which was found to be non-significant (*p* value of 0.747). Comparing mean labelling index of PD-L1 positive TAFs between OSCC and oral leukoplakia, higher expression was seen in oral leukoplakia compared to oral squamous cell carcinoma. The mean difference was found to be statistically significant (*p* value 0.000). The mean SID score of PD-L1 positive TAFs in oral leukoplakia was slightly higher compared to oral squamous cell carcinoma; however, this was found to be statistically non-significant (Tables 5 and 6).

**Table 5:** Comparison of PD1/PD-L1 between OSCC and oral leukoplakia.

Marker	Expression	Number of samples	OSCC	Oral leukoplakia	Chi square (df)	*p* value
PD-L1	No staining	N (%)	1 (4%)	20 (80%)	29.63 (1)	**< 0.001**
	staining present	N (%)	24** (**96.0%)	5 (20%)		
PD-L1 TAF	No staining	N (%)	6 (24%)	7 (28%)	0.104 (1)	0.747
	staining present	N (%)	19 (76%)	18 (72%)		
PD-1	No staining	N (%)	6 (24%)	3 (12%)	1.22 (1)	0.269
	staining present	N (%)	19 (76%)	22 (88%)		

Significant values are in bold.

**Table 6:** Comparison of various parameters between OSCC and oral leukoplakia.

Variable	Groups	N	Mean	Std. deviation	Std. error mean	Difference in means (confidence interval)	t statistic	*p* value
Labelling index of PD-L1 positive TAFs	OSCC	25	5.28	6.554	1.311	− 18.920 (− 28.56 to − 9.28)	− 4.022	**0.000**
	Oral leukoplakia	25	24.20	22.591	4.518			
SID score of PD-L1 positive TAFs	OSCC	25	1.80	0.764	0.153	− 0.160 (− 0.858 to 5.38)	− 0.465	0.645
	Oral leukoplakia	25	1.96	1.541	0.308			
Labelling index of PD-1 positive T-lymphocytes	OSCC	25	35.84	25.534	5.107	3.760 (− 10.941 to 18.461)	0.514	0.609
	Oral leukoplakia	25	32.08	26.164	5.233			
H score of PD-1 positive T-lymphocytes	OSCC	25	72.84	58.312	11.662	1.40 (− 34.460 to 37.260)	0.078	0.938
	Oral leukoplakia	25	71.44	67.467	13.493			

Significant values are in bold.

### Inter group comparison of expression of PD-1 among OSCC and OL

On comparing PD-1 positivity in inflammatory T-lymphocytes between oral leukoplakia and OSCC, PD-1 positivity was found higher in oral leukoplakia but was statistically not significant (*p* value of 0.269). Comparing the labelling index of PD-1 positive inflammatory T-lymphocytes between OSCC and oral leukoplakia, the mean labelling index was found to be higher in oral squamous cell carcinoma. Though statistically non-significant, the mean H score was higher in OSCC compared to oral leukoplakia (Tables 5 and 6).

### PD-L1 and PD1 expression in NOM

**Figure 3:**
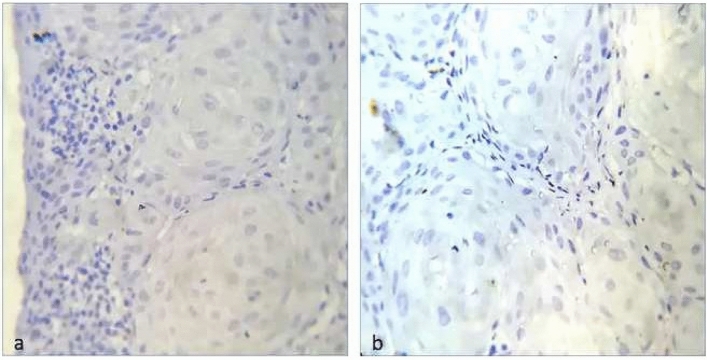
(**a**) Photo micrograph showing immunonegativity for PD-L1 in Normal Oral Mucosa. (**b**) Photo micrograph showing immunonegativity for PD-1 in Normal Oral Mucosa.

PD-L1 and PD-1 showed no immunoreactivity in normal oral mucosa (Fig. 3a,b).

## Discussion

### Cancer immune editing and their role in Precancerous lesions

Cancer immunoediting has been proposed as a potential mechanism by which tumours escape the host immune system, and it represents one of the currently accepted hallmarks of cancer^19,22^. It comprises three phases: elimination (tumour eradication), equilibrium (tumour dormancy), and escape (tumour outgrowth in an uncontrolled manner). Progression of premalignant lesions like oral leukoplakia to malignant OSCC is being intensely researched. Oral leukoplakia comprises histologically of dysplastic epithelial cells which are restricted to the epithelium. It has been hypothesized that the precancerous lesion may appear in the equilibrium phase of cancer immunoediting, in which the adaptive immune system cannot destroy the lesion, but prevents stromal invasion of the dysplastic epithelial cells. Additionally, the malignant transformation of precancerous lesion may equal the progression of lesion development from the equilibrium to the escape phase. As one of the major mechanisms of tumour escape, the establishment of an immunosuppressive tumour microenvironment through the induction of adaptive immune resistance by the activation of PD-1/PD-L1 inhibitory checkpoints has been highlighted recently. However, their role in the tumour microenvironment of oral precancerous lesions and the mechanisms activated by this environment in their malignant transformation has not been clarified^19^.

### PD-L1/PD-1 pathway and oral leukoplakia

We observed PD-L1 expression on both epithelium and subepithelial cells to examine whether an increased number of TAFs is recruited to the superficial lamina propria with the increase in the degree of epithelial dysplasia. TAFs recruitment may be induced by dysplastic epithelial cells through the secretion of growth factors and chemokines. Recent reports have implicated the expression of immunoinhibitory checkpoints, such as PD-1 expression on TILs, as the molecular mediators of an immunosuppressive tumour microenvironment^23^. When PD-L1 binds PD-1, which is present on the surface of TILs, these lymphocytes become inactivated. The expression of PD-L1 in the tumour microenvironment endows tumours with a mechanism to evade eradication by the host immune system^19,24,25^. Immunomodulatory human/humanized monoclonal antibodies, which target the PD-1/PD-L1 pathway, have demonstrated durable cancer control in clinical trials. PD-L1 expression in both epithelial and subepithelial cells are associated with malignant transformation. This suggests that anti-tumour immunity is suppressed through the PD-L1 expression on subepithelial cells as well as dysplastic epithelial cells, causing malignant transformation of oral precancerous lesions.

### Study findings about PD-L1/PD-1 pathway in oral leukoplakia

In present study PD-L1 positivity was noted both in dysplastic epithelial and subepithelial TAFs cells. We could infer that there are multiple PD-L1-expressing cell types within the tumour microenvironment of oral precancerous lesion. In case of mild dysplasia only single case showed basal epithelial staining while four cases showed PD-L1 positivity at subepithelial TAFs. Hence, most of the cases of mild dysplasia were PD-L1 negative. The moderate/severe dysplasia specimen showed PD-L1 expression on both basal epithelial and subepithelial TAFs cells. The SID score of moderate dysplasia was higher compared to mild dysplasia and was found to be statistically significant. This suggests that an increased number of TAFs is recruited to the superficial lamina propria occur with increase in the degree of epithelial dysplasia. Thus, TAFs could be considered to be important cells in the tumour microenvironment, which might play a key role in the progression of epithelial dysplasia.

The PD-1 expression was found to be higher in moderate dysplasia with a high H score compared to mild dysplasia. The labelling index of PD-L1 in TAFs along with labelling index of PD-1 in TILs were found to be non-significant in our study. About 68% cases of oral leukoplakia in our study exhibited both PD-L1 and PD-1 expression, which provide a new interpretation of precancerous lesions in terms of tumour immunology, showing that these lesions represent the equilibrium phase as defined by the concept of cancer immunoediting. The suppression of anti-tumour immunity in precancerous lesions through the PD-1/PD-L1 pathway may cause cancer progression from the equilibrium to the escape phase, causing the invasion of dysplastic epithelial cells into stromal tissue or malignant transformation. Furthermore, our hypothesis may explain the spontaneous disappearance of oral leukoplakia which has been previously observed and reported in the literature^26,27^, as the reverse transition from the equilibrium to the elimination phase when the anti-tumour immunity is enhanced or restored. In the elimination phase, the immune systems detect the lesions and destroy them, which results in the disappearance of oral precancerous lesions. Previous studies have suggested that the removal of instigating factors may have led to the restoration of anti-tumour immunity, was linked to the disappearance of oral leukoplakia^19,27,28^.

Shankaran et al.^29^ postulated cancer immunoediting concept in 2001, highlighting the importance of the identification of the tumor antigens expressed in the early phase of cancer development, because these antigens represent the initial targets of the elimination phase^30^. However, almost all tumor antigen studies are based on the analyses of advanced cancers belonging to the escape phase, because it is difficult to observe early-phase cancers, where the immune system usually destroys the lesion before it is clinically apparent. As previously explained, if spontaneous disappearance of oral leukoplakia is related with the enhancement or restoration of anti-tumor immunity, we can observe the elimination phase of cancer development in patients with oral leukoplakia, which is a commonly encountered oral precancerous lesion and it may represent a new target in tumor immunology research. The main purpose of identifying oral precancerous lesions is to prevent their further malignant transformation.

### PD-L1/PD-1 pathway and OSCC

In case of OSCC, PD-L1/PD-1 interactions causing negative regulation of activated T-cells have been established through several in vitro and in vivo studies. Although the entire mechanism is not completely understood, such inhibition is thought to be linked to the phosphorylation of the cytoplasmic domain of PD-1 and subsequent recruitment of phosphatase SHP-2.

### Study findings about PD-L1/PD-1 pathway in OSCC

In the present study about 96% cases of OSCC exhibit tumour epithelial PD-L1 positivity and 76% of cases exhibit positivity in TAFs. On comparing the expression with different grades of OSCC, no statistical significance was observed, it may be because of small samble size. The SID score of PD-L1 positive TAFS in our study was found higher in well differentiated OSCC compared to moderate/poorly differentiated carcinomas. Hence, as a component of the tumour microenvironment, along with TIL, TAFs can influence tumour growth by producing growth factors and chemokines. When expressing PD-L1, TAFs are expected to inhibit the function of TILs^17^. Many studies have indicated that higher PD-L1 expression correlates with poor prognosis^31^.

PD-1 expression in the present study was found to be slightly higher in moderate/poorly differentiated than well differentiated OSCC. However, H score was slightly higher in well differentiated than moderate/poorly differentiated OSCC. While corelating labelling index and H- score of PD-L1 positive tumour epithelial cells with labelling index and H-score of PD-1 positive TILs, we could obtain statistically significant results (*p* value of 0.005 and 0.001).Correlating the PD-L1/PD-1 positivity, we could infer that T-cell inactivation occurs in the microenvironment at the invasive front and is possibly involved in cancer progression.

### Study findings about comparisson of PD-L1/PD-1 pathway in oral leukoplakia and OSCC

Comparing PD-L1 and PD-1 positivity between OSCC and oral leukoplakia, a statistically significant mean labelling index of subepithelial PD-L1 positive TAFs was noted in oral leukoplakia. The sub epithelial PD-L1 positive TAFs cells were higher in oral leukoplakia compared to OSCC. Considering the novelty, on literature search only two studies have^7,32^ compared PD-L1 in oral leukoplakia to OSCC lesions, therefore it was difficult to compare the significance of immune checkpoint molecule expression between these disease states. Chen et al.^7^ assessed PD-L1 expression between OSCC and oral leukoplakia and determined that expression was greater in OSCC than oral leukoplakia , using a different scoring system compared to present study thus inferring a relative abundance of PD-L1 to be an indicator of disease progression. Another study by Malaspina TS et al.^30^ compared expression of PD-1 and PD-L1 in patients with actinic cheilitis and oral squamous cell carcinoma. There is a significant gap in the literature pertaining to the comparative expression of PD-1/PD-L1 biomarkers in OSCC, oral leukoplakia and to normal mucosa. There is a need to standardize detection methods, evaluation and the classification of positive expression across different tumour types and subsites within the oral cavity, in order to make the data less ambiguous. Despite the equivocal nature of current evidence, there is support for the prognostic and predictive values of immune checkpoint molecules, especially PD-1/PD-L1, and many studies provide support for the effective use of immune checkpoint inhibitors in the management of OSCC. Limited data is available for oral potentially malignant disorders (OPMD), therefore this should be the focus of future research.

## Data Availability

All data underlying the results are available as part of the article and no additional source data are required.
